# The Cumberland Ankle Instability Tool (CAIT) in the Dutch population with and without complaints of ankle instability

**DOI:** 10.1007/s00167-016-4350-4

**Published:** 2016-10-06

**Authors:** Gwendolyn Vuurberg, Lana Kluit, C. Niek van Dijk

**Affiliations:** Department of Orthopaedic Surgery, Orthopaedic Research Center Amsterdam, Academic Medical Centre, PO Box 22660, 1100 DD Amsterdam, The Netherlands

**Keywords:** Validity, Cumberland Ankle Instability Tool, Instability, Questionnaire, PROMS, Ankle

## Abstract

**Purpose:**

To develop a translated Dutch version of the Cumberland Ankle Instability Tool (CAIT) and test its psychometric properties in a Dutch population with foot and ankle complaints.

**Methods:**

The CAIT was translated into the Dutch language using a forward–backward translation design. Of the 130 subsequent patients visiting the outpatient clinic for foot and ankle complaints who were asked to fill out a questionnaire containing the CAIT, the Foot and Ankle Outcome Score (FAOS), and the numeric rating scale (NRS) pain, 98 completed the questionnaire. After a 1-week period, patients were asked to fill out a second questionnaire online containing the CAIT and NRS pain. This second questionnaire was completed by 70 patients. With these data, the construct validity, test–retest reliability, internal consistency, measurement error, and ceiling and floor effects were assessed. Additionally, a cut-off value to discriminate between stable and unstable ankles, in patients with ankle complaints, was calculated.

**Results:**

Construct validity showed moderate correlations between the CAIT and FAOS subscales (Spearman’s correlation coefficient (SCC) = 0.36–0.43), and the NRS pain (SCC = −0.55). The cut-off value was found at 11.5 points of the total CAIT score (range 0–30). Test–retest reliability showed to be excellent with an intraclass correlation coefficient of 0.94. Internal consistency was high (Cronbach’s *α* = 0.86). No ceiling or floor effects were detected.

**Conclusion:**

Based on the results, the Dutch version of the CAIT is a valid and reliable questionnaire to assess ankle instability in the Dutch population and is able to differentiate between a functionally unstable and stable ankle. The tool is the first suitable tool to objectify the severity of ankle instability specific complaints and assess change in the Dutch population.

*Level of evidence *II.

## Introduction

Self-reported outcome instruments or patient-reported outcome measures (PROMs) are gaining popularity. This is mainly due to the importance of monitoring the subjective effectiveness of received treatments, required in the current healthcare system to assess treatment quality [16, 28]. Currently, treatment results of ankle instability are more and more often evaluated using PROMs in combination with the traditionally used radiographs or manual tests, performed by the physician [16, 26]. The outcome of objective tests does not necessarily correspond with subjective feelings of patients [26], whereas PROMs provide feedback on patients’ view of their complaints. They combine efficiency with reliability and low costs [6].

A common injury that may be assessed using PROMs is ankle sprains, which are the most common sports injury. Up to 30 % of people who suffer from an initial ankle sprain experience persisting symptoms, which can progress to chronic ankle instability (CAI) [1, 24, 27]. CAI can lead to a wide spectrum of disabilities, e.g. loss of mechanical restraint, and recurrent sprains, requiring treatment [12].

A large number of self-reported outcome measures have been developed to assess foot and ankle complaints [3, 7, 9, 10, 13, 17, 20, 22, 24]. Of these, the Foot and Ankle Outcome Score (FAOS) and Foot and Ankle Ability Measure (FAAM) have been validated for assessing functional disability in patients with (chronic) ankle instability. The FAOS and FAAM have also been validated for the Dutch language [10, 21, 24, 26, 28]. They, however, are not specific for symptoms of instability. They do not take recurrent sprains or feelings of giving way, into account, even though it may be speculated that these symptoms may be the main cause of disability.

Hiller et al. [13] designed the Cumberland Ankle Instability Tool (CAIT). It was originally developed in English and proved to be of high content validity and good reliability. The main advantage of the questionnaire is that it consists of only 9 items, minimizing patient burden and increasing reliability. The precision of the instrument is increased as it is a multiple answer option instrument. In contrast to some other ankle instability questionnaires, like the Ankle Instability Instrument (AII), the CAIT is able to measure the severity of instability using a numeric value [4]. The CAIT is filled out for both the left and right ankle, making it possible to assess both ankles individually.

The CAIT has already been validated in English, Brazilian-Portuguese, Spanish and Korean [4, 6, 13, 15]. However, up to date there is no validated Dutch version of the CAIT available, nor other questionnaires assessing instability specific complaints. To be able to use the CAIT to assess the severity of ankle instability and assess complaints over time, the purpose of this study is a cross-cultural translation and an adaptation of the CAIT into the Dutch language and to validate the CAIT in the Dutch population. It is hypothesized the CAIT is a reliable and valid questionnaire usable to objectify (chronic) ankle instability in the outpatient clinic.

## Materials and methods

### Cross-cultural adaptation

Cross-cultural adaptation and validation of the Dutch version of the CAIT was performed according to the guidelines by Beaton et al. [2] using forward–backward translation. Translation from English to Dutch was performed independently by two bilingual translators (native Dutch-speaking orthopaedic researchers in the field of foot and ankle surgery). The two versions of the CAIT were compared, and a single consensus version was developed. Back-translation from Dutch into English was performed by a, not medically schooled, native English speaking translator, blinded to the original English version of the CAIT with the aim of preserving the meaning of items. The original and back-translated version was compared with the original CAIT. Only minor differences were found with no change in the meaning of the questions. Thus, a final Dutch version was created.

### Study outline

All consecutive native Dutch-speaking patients with foot and ankle complaints visiting the orthopaedic outpatient clinic of the Academic Medical Centre (AMC) in Amsterdam were asked to participate. Patients were excluded if they had an ankle arthrodesis or if they wore a plaster cast. This population was chosen to approach the normal patient population that would fill out this questionnaire at the orthopaedic outpatient clinic. Informed consent was verbally obtained before patients were handed the questionnaire. Patients were included if they were native Dutch-speaking and at least 12 years old. Patients were excluded if they did not fill out 2 or more questions of the CAIT to minimize interpretation bias. If a question was left open/blank, it was substituted with the mean questionnaire score.

After 7 days, patients were sent a second questionnaire by email, containing the numeric rating scale (NRS), the CAIT, and an additional question to measure change in complaints over the week (containing the options “much worse”, “worse”, “no change”, “better”, “much better”). To increase response rates, reminders were sent three times over a 1-week period. In case email addresses were not available, the second questionnaire was sent to the patients’ home address. The time limit for filling out the second questionnaire was 1 week after the last reminder.

### Outcome measures

The questionnaire inquired on demographic details and ankle complaints, including self-reported ankle instability to assess whether patients regarded themselves as functionally stable or not. Furthermore, it contained the CAIT, NRS pain, FAOS questionnaires and a comment box. The second questionnaire additionally contained a question to detect changes in complaints.

The CAIT is a 9-item scale measuring the severity of functional ankle instability (see Appendices 1 and 2 for the original and the Dutch version). The total score ranges from 0 to 30. Items focus on the degree of difficulty in performing different physical activities per ankle. The CAIT has the ability to discriminate between stable and unstable ankles and measures the severity of experienced functional instability, with a cut-off value of 27.5 points according to Hiller et al. [13].

The FAOS is a questionnaire that contains 5 subscales and intends to evaluate the functional limitation and symptoms caused by foot or ankle problems [21, 26].

The NRS was used to rate pain in rest, during walking, running and practicing sports on an 11-point rating scale, ranging from 0 (no pain) to 10 (worst pain imaginable).

### Psychometric properties

#### Validity

##### Construct validity

Due to the lack of a “gold standard”, validity was assessed in terms of consistency of the CAIT in relation to the FAOS and NRS, which have already been validated, and complaints of ankle instability [25]. It was hypothesized that lower scores on the FAOS subscales, and higher scores on the NRS pain scale, correlate with lower scores on the CAIT. Construct validity was evaluated using Spearman’s correlation coefficients. The correlation coefficient (CC) was considered poor when ≤0.30, moderate between 0.30 and 0.60, and strong when >0.60 [14, 25]. To assess whether the CAIT or the FAOS is more suitable to use for patients with ankle instability, the correlation between the CAIT and self-reported ankle instability, and the FAOS and self-reported ankle instability was assessed. Hypothesized was that the CAIT score would have a higher correlation with the self-reported ankle instability when compared to the FAOS sub-scales.

##### Cut-off value

Hiller et al. [13] calculated a cut-off value of 27.5 as an indication for an unstable ankle in their design of the CAIT. This means all scores of ≤27 represent unstable ankles, and only 3 points (28–30 points) represent a stable ankle. This value was obtained using previously reported ankle sprains as the baseline for ankle instability. To increase the accuracy of determining a cut-off value, this study chose self-reported ankle instability as the cut-off factor to reflect the CAIT score. To determine the cut-off value, a receiver operating characteristics (ROC) curve was used to find the highest Youden index [23].

#### Reliability

##### Test–retest reliability

Test–retest reliability was assessed on the subgroup which filled out the CAIT twice with a 1-week interval and additionally reported no difference in complaints. It was determined with the intraclass correlation coefficient (ICC) [16]. Reliability was considered poor if ICC was ≤0.40, moderate between 0.40 and 0.75, substantial between 0.75 and 0.90, and excellent >0.90 [25].

##### Internal consistency

The internal consistency was determined using the Cronbach’s *α* [25]. The CAIT was considered internally consistent when the items correlated moderately with each other and correlated moderately with the total score (Cronbach’s *α* = 0.70–0.95). Additionally, the Cronbach’s *α* was calculated for the questionnaire in case a question would be removed, to see if a question negatively influenced the Cronbach’s *α* [16, 25].

##### Measurement error

Measurement error was calculated as the standard error of measurement (SEM); the square root of the within-subject variance using the test–retest reliability (SEM = SD·√(1−ICC) [5]. From the SEM, the minimal detectable change (MDC) was calculated at an individual level (MDC_individual_ = 1.96·√2−SEM) and group level (MDC_group_ = MDC_individual_/√*n*) [25].

#### Interpretability

##### Ceiling and floor effects

Ceiling and floor effects were defined as being present if more than 15 % of the participants had a final score equal to the highest respectively lowest total score possible [25].

### Statistical analysis

Data were analysed for normality using the Shapiro–Wilks test. Skewed data are presented with a median and range, and continuous data are presented with a mean and standard deviation. A p-value of <0.05 was considered statistically significant. Psychometric properties, as described above, were analysed using IBM SPSS 22.0 (Chicago, Illinois, USA).

### Methods

This study was conducted in line with the Declaration of Helsinki and received approval of the Institutional Review Board (IRB) of the Academic Medical Center of Amsterdam. The requirement for informed consent was waived by the local IRB. ID number: W15_214 # 15.0254.

## Results

Of 130 patients that responded to the initial invitation to participate in this study, 98 (75 %) completed the questionnaire. From these, 70 patients (71 %) filled out the second questionnaire. Of the first questionnaire, 45 % of patients were female, and 55% were male. Ages ranged from 16 to 74 with a median age of 40 years. Fifty-five patients (56 %) reported complaints of ankle instability.

### Validity

#### Construct validity

Both the FAOS subscales and the NRS pain showed significant correlations with the total CAIT score. These correlations were of moderate value. A high negative correlation was found between complaints of ankle instability and the total CAIT score (Table 1). Assessing the correlation between the FAOS subscales and self-reported ankle instability showed no significant correlations (*p* = 0.051–0.186).

**Table 1:** Correlation between the total CAIT score and the NRS and FAOS

Question/score	Correlation with the CAIT	*p* value
Self-reported instability	−0.65	<0.0005
NRS pain scale	−0.55	<0.0005
FAOS pain	0.42	<0.0005
FAOS symptoms	0.37	<0.0005
FAOS ADL	0.48	<0.0005
FAOS sport	0.36	<0.0005
FAOS QoL	0.43	<0.0005

*ADL* Activities of daily living, *CAIT* Cumberland Ankle Instability Tool, *FAOS* Foot and Ankle Outcome Score, *NRS* numeric rating scale, *QoL* quality of life

#### Cut-off value

**Fig. 1:**
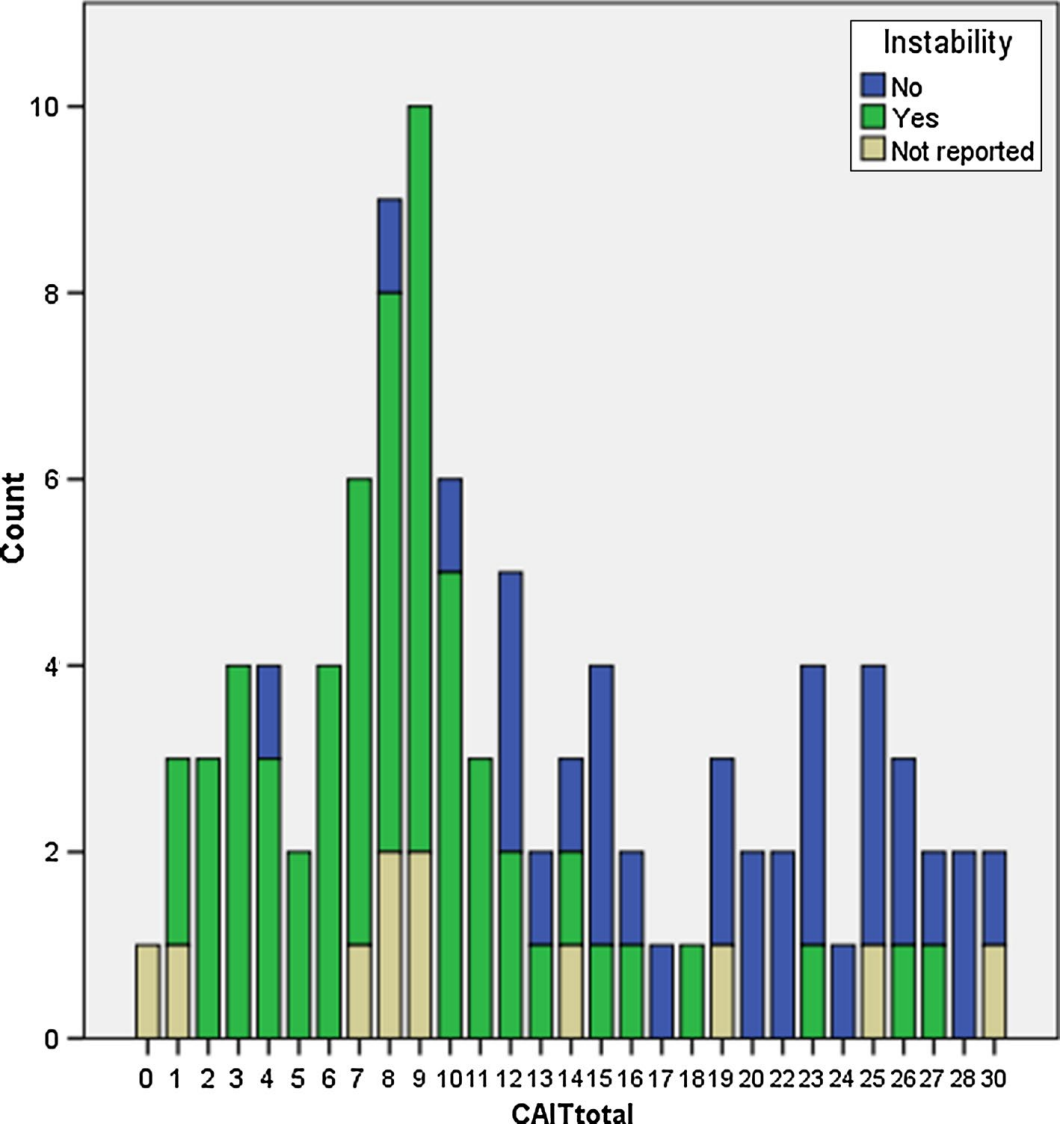
Patient count per CAIT score categorized by patients with (Yes) and without (No) self-reported ankle instability

Of patients who reported complaints of ankle instability, 85.5 % (*n* = 47) scored a maximum of 12 points, with only 8 patients scoring ≥13 points, whereas of patients who did not report complaints of ankle instability, 93 % scored above 12 points (*n* = 29) with 2 outliers scoring 4 and 8 points (Fig. 1). This suggests the cut-off value lies near 12 points. The Youden index was highest at 11.5-points, resulting in the cut-off value between functional ankle instability ≤11 or a stable ankle ≥12 (Table 2).

**Table 2:** Sensitivity and specificity of the CAIT to determine the Youden index and concurrent cut-off value

CAIT score	9.50	10.50	11.50	12.50	13.50
Sensitivity	0.67	0.76	0.82	0.86	0.87
Specificity	0.94	0.91	0.91	0.81	0.78
Youden index	0.61	0.67	0.72	0.67	0.65

### Reliability

Test–retest reliability could only be measured for 78% (*n* = 55) of patients that filled out both the first and second questionnaire, as they reported no change over a 1-week period. Of this subgroup, 51 % were female and 49 % were male, the median age was 40 years, and 35 patients (64 %) reported complaints of ankle instability. The reliability between two measurements proved to be excellent with an ICC of 0.943. Internal consistency of the CAIT was also high with a Cronbach’s *α* of 0.856. The inter-item correlation between questions ranged from 0.5 to 0.7, except for question 1 (pain) and questions 8 and 9 (recovery after a sprain). The SEM proved to be 2.7 % (0.82/30) of the total score, with a relatively high MDC at an individual level, but a low MDC at a group level (Table 3).

**Table 3:** Reliability and interpretability of the CAIT shown as mean measurement, SEM and MDC

	First measurement mean (SD)	Second measurement mean (SD)	Mean difference (SD)	SEM	MDC_individual_	MDC_group_
CAIT	12.35 (7.60)	12.85 (7.26)	0.51 (3.6)	0.82	2.28	0.04

*MDC* Minimal detectable change, *SD* standard deviation, *SEM* standard error of measure

### Interpretability

No signs of ceiling or floor effects were detected, as only 1 patient (1 %) scored the minimal score of 0 points and 2 patients (2 %) scored 30 points. Interpretability was good as the SEM was low, and at an individual level only 3 points were needed to detect change, at a group level only 1 point was needed to detect change.

## Discussion

The most important finding of the current study was the new cut-off value to differentiate between a stable and unstable ankle. The Dutch version of the CAIT also showed moderate validity and high reliability. There is a significant (moderate) correlation between the CAIT and the FAOS, and the CAIT and NRS scores. The CAIT score also showed a (good) significant correlation with self-reported ankle instability. Internal consistency was high with an excellent test–retest reliability and no ceiling or floor effects. A cut-off value of 11.5 points was calculated using the Youden index. These results suggest that this translated version of the CAIT is suited for use in the Dutch population to assess complaints of ankle instability.

The FAOS and NRS were already available to the Dutch population. The FAOS has also been previously validated for patients with ankle instability. Therefore, these were the most appropriate instruments to measure the construct validity of the CAIT [19, 21]. Furthermore, using only the FAOS and the NRS to measure construct validity minimized patient burden.

Previous validation studies by Cruz-Diaz et al. [4] and Ko et al. [15] used the SF-36, the Visual Analogue Scale, and the Lower Extremity Functional Scale to assess construct validity. Cruz-Diaz et al. found a CC of the CAIT with the SF-36 physical component of 0.241, whereas Ko et al. found a CC with the physical component of the SF-36 of 0.70 [4, 15]. Differences to the Spearman’s CC in this study may have been caused by the different measurement tools used, smaller or larger sample sizes or a more heterogeneous group of participants (wider range in age, no healthy volunteers, different ratio of participants with and without ankle instability). Comparing the Spearman’s CC’s within this study, showed that self-reported instability has a strong correlation with the CAIT score, whereas the correlations with the FAOS and the NRS were only moderate. Therefore we additionally analysed the correlation of the FAOS with self-reported instability, showing no significant correlation with self-reported instability. This may indicate that the CAIT is more suited to evaluate ankle instability than the FAOS. The second highest correlation found was the NRS pain, which has a moderate negative correlation with the CAIT score.

The two peaks in Fig. 1 represent two populations participating in this study: patients with and without self-reported ankle instability. The cut-off value of 11.5 points, calculated using the Youden index, is located in between these two peaks. In contrast to this cut-off value, Hiller et al. [13] described 27.5 points as the cut-off value. This value was later recalculated by Wright et al. [29] and set at ≤25 points. However, a patient with a highly painful ankle due to an unrelated cause could be concluded to have functional ankle instability with these cut-off values, based only on a low score on the first question. Thus in a clinical setting, with patients with foot or ankle complaints, these cut-off values would be inapplicable. The great contrast with the 11.5 points as cut-off value, found in this study, may be due to the fact that both Hiller et al. [13] and Wright et al. [29] based their cut-off value on a history of an ankle sprain. A sprain is, however, very common in the general population and does not lead to ankle instability in the majority of the cases [8, 24, 27]. In this study we chose to use self-reported ankle instability to calculate a cut-off value instead. This, as the questionnaire aims to evaluate, reflects ankle instability as experienced by the patient (their subjective complaints) [11]. The aim of determining the cut-off value was to find out which CAIT scores indicate unstable ankles by matching this to self-reported ankle instability. If patients report complaints of ankle instability, this should become visible in their CAIT score compared to patients that report no ankle instability. This cut-off value only indicates whether patients experience ankle instability (functional instability). To determine a cut-off value for mechanic instability, physical examination should be combined with the validation of the questionnaire. Combining the CAIT with physical examination may help define the best treatment. Furthermore, Wright et al. [29] recruited relatively healthy individuals from a metropolitan area including a university campus, who may not seek help for their complaints. The difference in these cut-off values exposes the issue of the amount of influence the chosen cut-off factor and the chosen target population may have. This study recruited only patients at the outpatient clinic with foot and ankle complaints, as this will be the target population that will fill out the questionnaire to evaluate their treatment, and is, therefore, in this case, the population in which the questionnaire had to be validated.

Test–retest reliability of the CAIT score was excellent with an ICC of 0.94. The ICC of other previous validations was greater than, or equal to 0.95 [4, 6, 13, 15]. In this case, only 78 % of patients reported no change in complaints. This may be due to the different moments the questionnaire was filled out and the different situation (at the outpatient clinic and at home) where the questionnaire was filled out, leading to a change in how complaints may have been experienced. The 22 % that reported a change in condition were excluded from this analysis. However, Hiller et al. [13] still showed excellent results with a time period of 2 weeks in between both questionnaires.

The results showed a Cronbach’s *α* of 0.86, concluding the internal consistency was similar to other studies validating the CAIT, ranging from 0.77 to 0.88 [4, 6, 13, 18]. As this was not highly correlated (Cronbach’s *α* 0.95), there is a low risk of items being redundant.

The SEM (0.82) was lower compared to the previous SEM calculated for the Korean validation (1.72). The minimally detectable change (MDC) of the CAIT score on the individual level was 2.28 points and at the group level (*n* = 55) 0.04 points. It must be noted that the CAIT measures subjectively experienced functional ankle instability. It was not possible to compare the MDC to other studies, as this study is the first to evaluate this for the CAIT [15]. However, in the case of an individual measurement, the MDC was low (7.6 % of the maximal score), at a group level, only one point in score change is needed to indicate a change in patient complaints. No harmful effects by means of a ceiling and/or floor effects were shown in this study or previous studies [4, 6, 18].

Results were limited, as not all subsequent patients visiting the outpatient clinic asked to fill out the questionnaire, participated. Additionally, only 75 % of the initially recruited patients completed the first questionnaire, and only 71 % completed the second questionnaire. This was due to the high amount of patients leaving more than 1 question open/blank and therefore being excluded. Another limitation may have been caused by the study population. The patients that filled out the questionnaire all suffered from some form of ankle/foot complaints. This may be the cause we did not find any floor or ceiling effects of the CAIT. However, the clinical setting was chosen on purpose, to test the CAIT among patients with severe ankle disability. This was done because the score will mainly be used to evaluate ankle instability pre- and post-operatively in patients with possible additional complaints. Finally, the self-reported ankle instability was not evaluated by a physician, unless patients visited the outpatient clinic specifically for instability complaints. To assess the severity of functional ankle instability, the CAIT is currently the best questionnaire that can be used in a short time frame, with easily interpretable results due to the newly calculated cut-off value.

## Conclusion

The Cumberland Ankle Instability Tool is a valid and reliable instrument fit to assess ankle instability among the Dutch population. Additionally, the CAIT may be a more reliable evaluation of ankle instability complaints compared to the FAOS because of the minimization of patient burden due to the lower amount of questions.

## Appendix 1: Original version of the Cumberland Ankle Instability Tool

Please tick the ONE statement in EACH question that BEST describes your ankles.

**Table:**

	Left	Right	Score
1. I have pain in my ankle			
Never	□	□	5
During sport	□	□	4
Running on uneven surfaces	□	□	3
Running on level surfaces	□	□	2
Walking on uneven surfaces	□	□	1
Walking on level surfaces	□	□	0
2. My ankle feels UNSTABLE			
Never	□	□	4
Sometimes during sport (not every time)	□	□	3
Frequently during sport (every time)	□	□	2
Sometimes during daily activity	□	□	1
Frequently during daily activity	□	□	0
3. When I make SHARP turns, my ankle feels UNSTABLE			
Never	□	□	3
Sometimes during running	□	□	2
Often when running	□	□	1
When walking	□	□	0
4. When going down the stairs, my ankle feels UNSTABLE			
Never	□	□	3
If I go fast	□	□	2
Occasionally	□	□	1
Always	□	□	0
5. My ankle feels UNSTABLE when standing on ONE leg			
Never	□	□	2
On the ball of my foot	□	□	1
With my foot flat	□	□	0
6. My ankle feels UNSTABLE when			
Never	□	□	3
I hop from side to side	□	□	2
I hop on the spot	□	□	1
When I jump	□	□	0
7. My ankle feels UNSTABLE when			
Never	□	□	4
I run on uneven surfaces	□	□	3
I jog on uneven surfaces	□	□	2
I walk on uneven surfaces	□	□	1
I walk on a flat surface	□	□	0
8. TYPICALLY, when I start to roll over (or “twist”) on my ankle, I can stop it			
Immediately	□	□	3
Often	□	□	2
Sometimes	□	□	1
Never	□	□	0
I have never rolled over on my ankle	□	□	3
9. After a TYPICAL incident of my ankle rolling over, my ankle returns to “normal”			
Almost immediately	□	□	3
Less than one day	□	□	2
1–2 days	□	□	1
More than 2 days	□	□	0
I have never rolled over on my ankle	□	□	3

## Appendix 2: Dutch version of the Cumberland Ankle Instability Tool

Kruis maximaal 1 vakje aan dat het meest past bij uw situatie.

**Table:**

	Links	Rechts	Score
1. Ik heb pijn in mijn enkel			
Nooit	□	□	5
Tijdens sport	□	□	4
Bij rennen op oneven ondergrond	□	□	3
Bij rennen op vlakke ondergrond	□	□	2
Bij lopen op oneven ondergrond	□	□	1
Bij lopen op vlakke ondergrond	□	□	0
2. Mijn enkel voelt ONSTABIEL			
Nooit	□	□	4
Soms tijdens sport (niet elke keer)	□	□	3
Vaak tijdens sport (elke keer)	□	□	2
Soms tijdens dagelijkse activiteiten	□	□	1
Vaak tijdens dagelijkse activiteiten	□	□	0
3. Als ik een SCHERPE draai maak, voelt mijn enkel ONSTABIEL			
Nooit	□	□	3
Soms tijdens rennen	□	□	2
Vaak tijdens rennen	□	□	1
Tijdens lopen	□	□	0
4. Bij het van de trap af gaan, voelt mijn enkel ONSTABIEL			
Nooit	□	□	3
Als ik snel ga	□	□	2
Af en toe	□	□	1
Altijd	□	□	0
5. Mijn enkel voelt ONSTABIEL bij het staan op ÉÉN been			
Nooit	□	□	2
Op de bal van mijn voet	□	□	1
Met mijn voet plat	□	□	0
6. Mijn enkel voelt ONSTABIEL wanneer…			
Nooit	□	□	3
Ik van links naar rechts hop	□	□	2
Ik op de plaats hop	□	□	1
Ik spring	□	□	0
7. Mijn enkel voelt ONSTABIEL als…			
Nooit	□	□	4
Ik ren op oneven ondergrond	□	□	3
Ik jog op oneven ondergrond	□	□	2
Ik loop op oneven ondergrond	□	□	1
Ik loop op vlakke ondergrond	□	□	0
8. TYPISCH, wanneer ik begin met mijn enkel verzwikken (of verstuiken) kan ik dit stoppen…			
Direct	□	□	3
Vaak	□	□	2
Soms	□	□	1
Nooit	□	□	0
Ik heb nog nooit mijn enkel verzwikt	□	□	3
9. Na een TYPISCH geval van mijn enkel verzwikken, wordt mijn enkel weer ‘normaal’…			
Bijna direct	□	□	3
Binnen één dag	□	□	2
1–2 dagen	□	□	1
Meer dan 2 dagen	□	□	0
Ik heb nog nooit mijn enkel verzwikt	□	□	3
